# Trends and findings of lipoprotein(a) testing and associated cardiovascular disease profiles: a large single-center study from the Middle East-Gulf region

**DOI:** 10.3389/fcvm.2024.1439013

**Published:** 2024-07-09

**Authors:** Yosef Manla, Laila AbdelWareth, Ronney Shantouf, Yazan Aljabery, Terrence Lee St John, Hani Sabbour, Bartlomiej Piechowski-Jozwiak, Wael Almahmeed

**Affiliations:** ^1^ Heart, Vascular and Thoracic Institute, Cleveland Clinic Abu Dhabi, Abu Dhabi, United Arab Emirates; ^2^ Pathology and Laboratory Medicine Institute, Cleveland Clinic Abu Dhabi, Abu Dhabi, United Arab Emirates; ^3^ National Reference Laboratory, Abu Dhabi, United Arab Emirates; ^4^ Khalifa University, Abu Dhabi, United Arab Emirates; ^5^ Research Department, Academic Office, Cleveland Clinic Abu Dhabi, Abu Dhabi, United Arab Emirates; ^6^ Neurological Institute, Cleveland Clinic Abu Dhabi, Abu Dhabi, United Arab Emirates

**Keywords:** cardiovascular disease, Middle East, hyperlipidemia, lipoprotein (a), metabolic syndrome

## Abstract

**Background:**

Lipoprotein(a) [Lp(a)] is a genetically determined risk factor for atherosclerotic cardiovascular disease (CVD). Limited data are available on Lp(a) testing from the Middle-East region. Therefore, we aim to evaluate the utilization and yield of Lp(a) testing over time and characterize CVD profiles of patients with abnormal Lp(a) tasting at a single-quaternary-care center in the United Arab Emirates.

**Methods:**

Unique Lp(a) tests conducted between 07/2017 and 10-2023 were included. Overtime trends in Lp(a) test utilization and abnormal Lp(a) [defined as Lp(a) > 125 nmol/L] test findings were described. CVD rates in patients with abnormal Lp(a) were compared to those with Lp(a) ≤ 125 nmol/L using appropriate methods.

**Results:**

In our center, 0.95% of the patients (*n* = 5,677) had their Lp(a) measured, with a median level of 32 [11–82] nmol/L. Lp(a) was abnormal in 15.9% of the tests. Over the years 2018–2022, there was a 109% increase in Lp(a) testing, with concomitant up-trends in findings of abnormal Lp(a) (11.8% to 16.4%, *P* = 0.02). Compared to patients with Lp(a) ≤ 125 nmol/I, those with abnormal Lp(a) had higher rates of any prevalent CVD (34% vs. 25.1%, *P* < 0.001), CAD (25.6% vs. 17.7%, *P* < 0.001), HF (6.5% vs. 3.8%, *P* < 0.001), and stroke (7.1% vs. 4.4%, *P* < 0.001).

**Conclusion:**

Almost one in six patients tested for Lp(a) had abnormally elevated Lp(a), and CVD was prevalent in one-third of the patients who tested abnormal for Lp(a). The study highlights the growing awareness of the relevance of Lp(a) for CVD risk stratification and prevention.

## Background

Lipoprotein (a) [Lp(a)] is a genetically determined, independent, and causal risk factor for atherosclerotic cardiovascular disease (CVD) (1, 2). Meta-analyses of prospective, population-based studies revealed a high risk of myocardial infarction (MI), coronary heart disease at Lp(a) concentrations above 62 nmol/L, and increased risk of ischemic stroke at Lp(a) concentrations above 100 nmol/L (1, 3–5). In addition, large prospective, population-based studies of high Lp(a) demonstrated that patients with the highest vs. lowest Lp(a) concentrations are at higher risk of MI, ischemic stroke, aortic stenosis (AS), carotid stenosis, heart failure (HF), CVD mortality, and all-cause mortality. Moreover, large Mendelian randomization studies further confirmed that increased Lp(a) is a causal factor for the aforementioned morbidities and mortality (3–10). Interestingly, these causal relationships were independent of concentrations of other lipids and lipoproteins, including low-density lipoprotein cholesterol (LDL-C).

The Middle East region features a high burden of CVD, CAD, stroke, and its associated cardio-renal-metabolic risk factors, as well as a high burden of heart failure (11–17). Limited data are available on Lp(a) testing from the Middle East Gulf region. In an analysis of 6,086 cases of first MI and 6,857 controls in the INTERHEART study, adjusted for age and sex and stratified by ethnicity, including 775 Africans and 1,352 Arabs (18), Lp(a) concentrations were highest in African and Arab cases. However, despite the differences in Lp(a) concentrations between ethnic groups, high Lp(a) concentration (defined as >50 mg/dl) was associated with MI overall (OR = 1.48) and across different ethnic subgroups, except for Africans and Arabs (18). Testing for Lp(a) has been recommended by guidelines and statements of major professional societies (1, 2, 10, 19–24). Reyes-Soffer et al. recently summarized these recommendations (2); all of these guidelines and statements recommend measuring Lp(a) in patients with personal and/or family history of premature atherosclerosis CVD. In addition, testing in individuals with moderate- to high risk of atherosclerotic CVD has also been recommended (1, 21, 24). The European Society of Cardiology (ESC)/European Atherosclerosis Society (EAS) recommended a universal measurement of Lp(a) at least once in a lifetime, which is also now recommended by the Canadian Cardiovascular Society, EAS, and was also included in a 2024 focused update to the scientific statement by the national lipids associations (NLA) (19, 20, 22, 23). This universal recommendation for an Lp(a) test is particularly important in the context of primary prevention and understanding the risk of CVD events in the absence of traditional risk factors (2). Studies from around the world highlighted increased testing trends of Lp(a) over the years. However, these rates remain low (25–29).

In this study of a single-quaternary care center in the United Arab Emirates, we aim to evaluate the utilization and yield of Lp(a) testing over time, describe levels of Lp(a) in patients with CVD vs. healthy individuals, and characterize CVD profiles of patients with abnormal Lp(a) testing.

## Methods

### Study design and definitions

This was a single-center retrospective cohort study conducted at Cleveland Clinic Abu Dhabi in the United Arab Emirates. Unique Lp(a) tests conducted since the initiation of in-house testing at our center (07-2017) until 10-2023 were included and described in this analysis. Lp(a) was measured using the Tina-quant® Lipoprotein (a) Gen. 2 assay. Data on baseline CVD profiles (including any diagnosis of the following: atrial fibrillation (AF), coronary artery disease (CAD), HF, peripheral vascular disease (PVD), Aortic Stenosis (AS), carotid stenosis, or stroke) and family history of CVD profiles of these patients using International Classification of Diseases-10 were collected retrospectively. Lp(a) test findings and abnormal Lp(a) [defined as Lp(a) > 125 nmol/I] were described for the overall population, as well as for patients with and without CVD. CVD profiles were compared between patients with abnormal Lp(a) testing vs. those with Lp(a) ≤ 125 nmol/I in the overall study population. For the included full years 2018–2022, annual trends in Lp(a) test utilization, abnormal Lp(a) test findings, and CVD profiles of tested patients were assessed. The study was reviewed and approved by the local Institutional Review Board (IRB), and informed consent was waived due to the deidentified nature of the data.

### Statistical analysis

The assumption of normal distribution was tested with the Shapiro-Wilk test. Continuous variables were presented as mean ± standard deviation and compared using t-tests (if normally distributed). Non-normally distributed continuous variables were presented as medians and inter-quartile ranges and compared using Wilcoxon rank-sum tests. Categorical variables were presented as frequencies and percentages and compared using a chi-square or Cochran-Armitage test, as appropriate. All comparisons were two-tailed, and *P*-values < 0.05 were considered statistically significant. Analysis was performed using JMP® Data Analysis (Software Version 17, SAS Institute Inc., Cary, NC, USA).

## Results

### Lp(a) testing findings and trends

**Figure 1:**
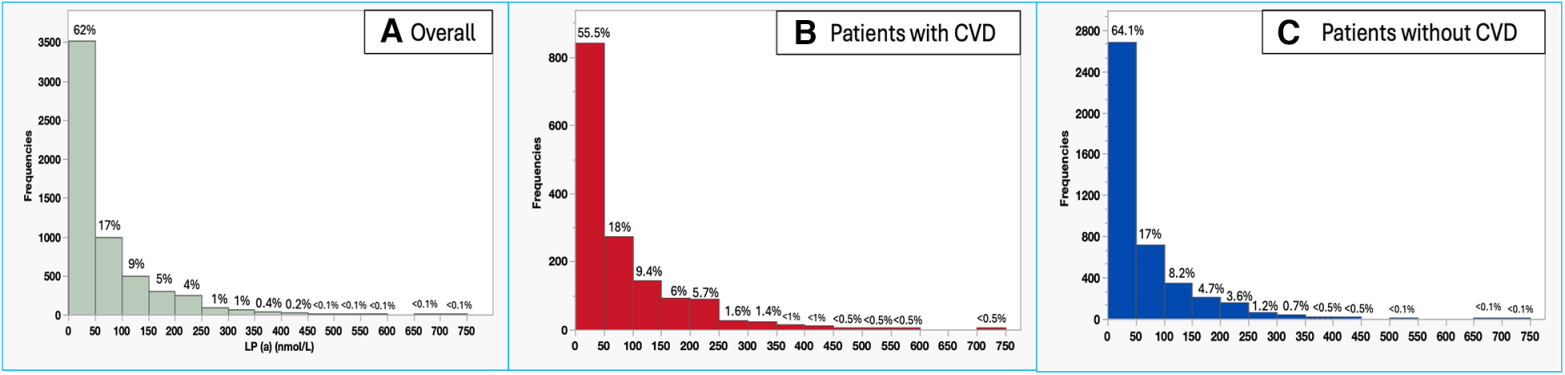
Histogram of Lp(a) levels among the overall tested population in our center (**A**), patients with CVD (**B**), and patients without CVD (**C**).

In our center, 0.95% (5,677/595,658) of the patients (mean age 50 ± 13 years, 62.4% males) had their Lp(a) measured during the study period, with a median level of 32 [11–82] nmol/I. Abnormal Lp(a) was evident in 15.9% (*n* = 903) of tests with a median of 190.9 [155–234.9] nmol/I. 62% of the patients had Lp(a) < 50 nmol/L, while 7.3% of the patients had Lp(a) ≥ 200 nmmol/L (Figure 1A).

**Figure 2:**
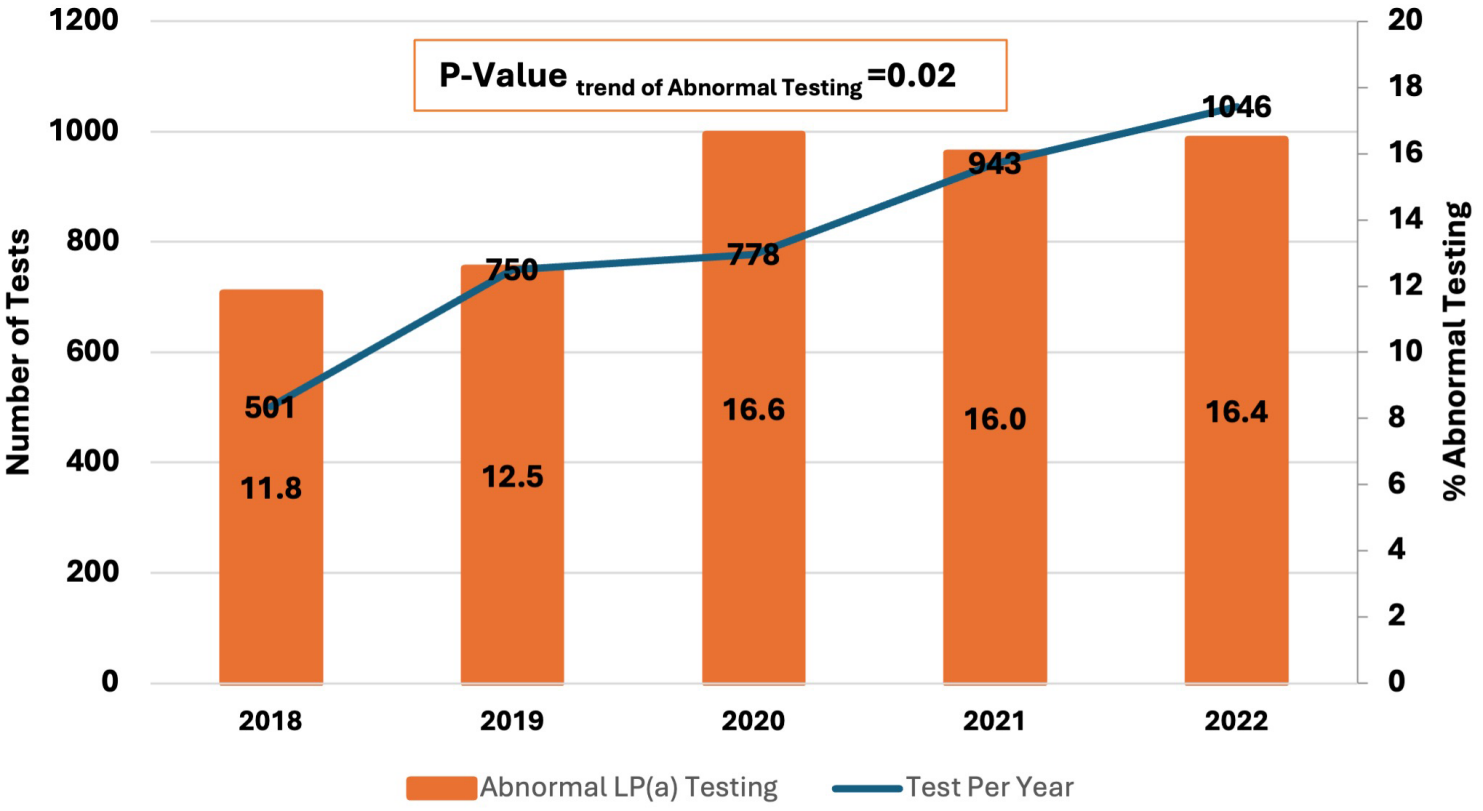
2018–2022 trends in Lp(a) testing and findings of abnormal Lp(a) [Lp(a) > 125 nmol/I] at a single center in the Middle East Gulf region.

**Figure 3:**
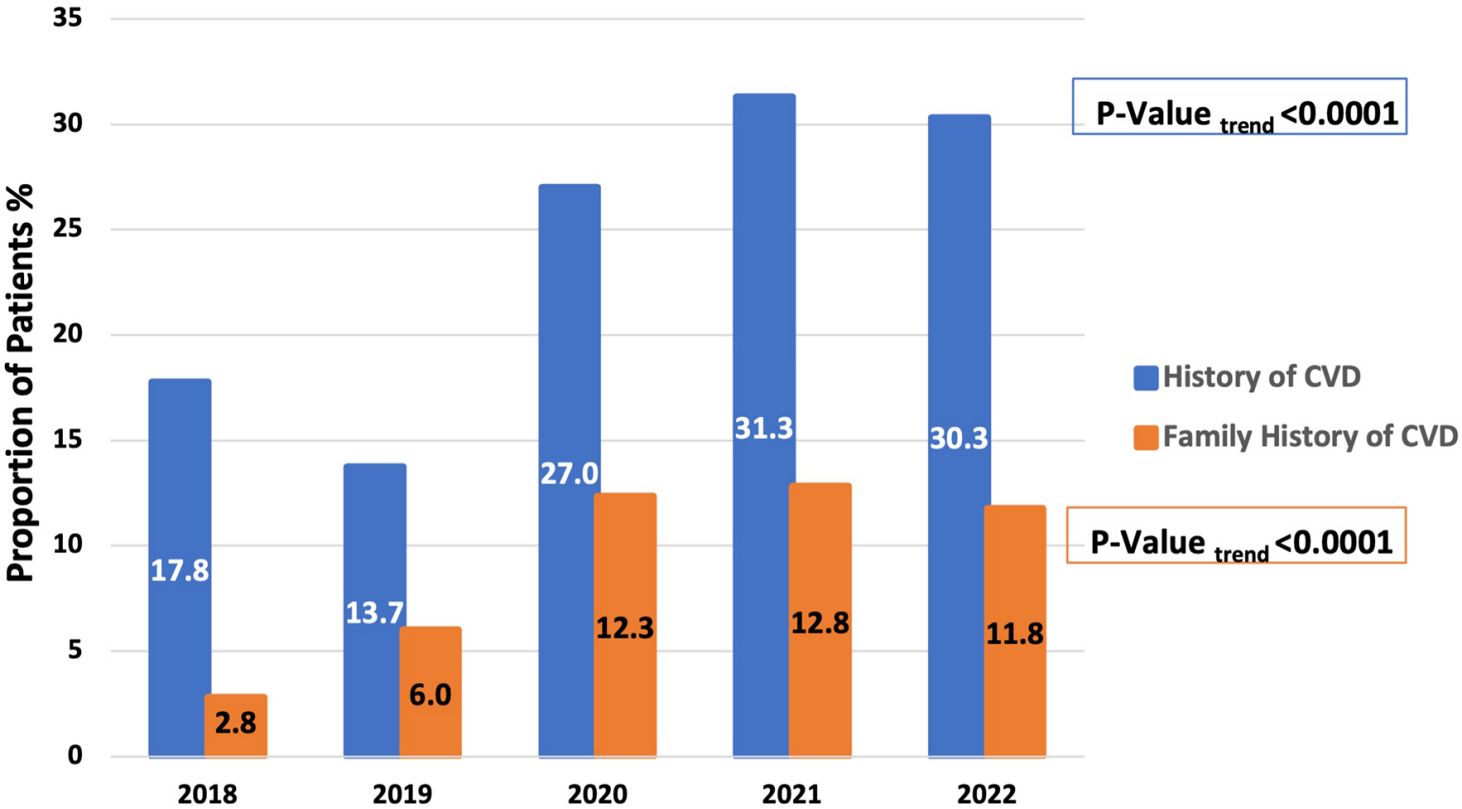
Increased representation of patients with a history of any cardiovascular disease or family history of cardiovascular disease among those tested for Lp(a) between 2018 and 2022 at a single center in the Middle East Gulf region.

When Studying trends in Lp(a) testing over the years 2018 to 2022, there was a 109% increase in Lp(a) test utilization at our center (501–1,046 tests per year), with a concomitant 40% up-trend in findings of abnormal Lp(a) test (11.8% to 16.4%, *P* = 0.02) (Figure 2). When studying the characteristics of tested patients over the years 2018–2022, there was a 70% increase in the proportion of patients with any CVD over time (17.8%–30.3%, Ptrend < 0.0001) and an increase of 321.4% in the proportion of patients with a family history of CVD (2.8%–11.8%, Ptrend < 0.0001) (Figure 3).

### Lp(a) levels in patients with CVD compared to healthy individuals

Among tested patients for Lp(a), those with CVD had approximately 40% higher median levels of Lp(a) as compared to healthy individuals (40 [14–105.9] vs. 28.6 [10.2–75] nmol/I, *P* < 0.001), with a higher proportion of patients with abnormal Lp(a) among patients with CVD (20.4% vs. 14.3%, *P* < 0.001). Figures 1B,C show the distribution of Lp(a) levels among patients with CVD and those without CVD (healthy individuals). Patients with CVD had a significantly lower proportion of patients with Lp(a) < 50 nmol/L (55.5% vs. 64.1%, *P* < 0.001) and a higher proportion of patients with Lp(a) ≥ 200 nmmol/L (11% vs. 5.9%, *P* < 0.001) as compared to healthy individuals.

### Cardiovascular disease profiles of patients with abnormal Lp(a)

Compared to patients with Lp(a) ≤ 125 nmol/I, those with abnormal Lp(a) had higher rates of any prevalent CVD (34% vs. 25.1%, *P* < 0.001), with a higher proportion of patients having at least 2 cardiovascular comorbidities (11.4% vs. 6.8%, *P* < 0.001). This included higher rates of CAD (25.6% vs. 17.7%, *P* < 0.001), HF (6.5% vs. 3.8%, *P* < 0.001), stroke (7.1% vs. 4.4%, *P* < 0.001), and carotid stenosis (3% vs. 1.7%, *P* = 0.02). However, no significant differences were recorded between groups in rates of family history of CVD (11.1% vs. 10.6%, *P* = 0.7), AF (2.8% vs. 3.1%, *P* = 0.8), AS (1.6% vs. 0.9%, *P* = 0.06) or PVD (3.8% vs. 2.8%, *P* = 0.1) (Table 1).

**Table 1:** Cardiovascular disease profiles of patients with Lp(a) ≤ 125 vs. Lp(a) > 125 nmol/I.

	Lp(a) ≤ 125 nmol/I (*n* = 4,774)	Lp(a) > 125 nmol/I (*n* = 903)	*P*-value
Family history of cardiovascular disease *n* (%)	508 (10.6%)	100 (11.1%)	0.7
History of any cardiovascular disease *n* (%)	1,200 (25.1%)	307 (34%)	**<0**.**001**
Coronary artery disease *n* (%)	845 (17.7%)	231 (25.6%)	**<0**.**001**
Heart failure *n* (%)	181 (3.8%)	59 (6.5%)	**<0**.**001**
Stroke *n* (%)	208 (4.4%)	64 (7.1%)	**<0**.**001**
Atrial fibrillation *n* (%)	146 (3.1%)	25 (2.8%)	0.7
Peripheral vascular disease *n* (%)	134 (2.8%)	34 (3.8%)	0.1
Aortic stenosis *n* (%)	41 (0.9%)	14 (1.6%)	0.06
Carotid stenosis *n* (%)	81 (1.7%)	27 (3%)	**0**.**02**

Bold values reflect statistically significant values.

## Discussion

In this single-center experience from the Middle East, we describe trends of Lp(a) testing, Lp(a) levels, and CVD profiles of the overall patient population. It is estimated that 1 in 5 people (≈1.5 billion) patients worldwide have an elevated Lp(a) (>125 nmol/L) (2); consistently, in our study, 15.9% of the tested patients had an abnormal Lp(a). Lp(a) is considered the most common inherited dyslipidemia as well as the strongest genetic risk factor for atherosclerotic CVD (2). This risk remains significant even in the absence of traditional risk factors or adherence to guideline-recommended LDL-C levels and lifestyle modifications (2). Therefore, it is essential for clinicians to implement the recommendation of Lp(a) measurement at least once in each adult person's lifetime for a comprehensive CVD risk assessment (2, 19, 20, 22, 23). However, Lp(a) testing rates remain low in clinical practice. Bhatia et al. reported in a large study of 6 academic health systems in California for the years (2012–2021), that only 0.3% of adults had Lp(a) testing (26). In a study of 4 million patient records in Germany, rates of Lp(a) testing ranged between 0.25% and 0.34% for the years 2015–2018 (25). In our study, we found that 0.95% of the patients receiving care in our center were tested for Lp(a) at least once during the study period, which is relatively higher but consistent with suboptimal rates from around the globe.

When analyzing trends of Lp(a) testing over the years 2018–2022, the number of tests per year increased by 109% (from 501 to 1,046). At our center, Lp(a) testing is ordered in the cardiology, neurology, and endocrinology clinics. Additionally, it has been incorporated into executive health and preventive medicine programs. There were also concomitantly increased rates of abnormal Lp(a) testing levels, which could be attributed to a greater representation of patients with a history of CVD or a family history of CVD among those who underwent Lp(a) testing. These findings highlight the increased awareness of Lp(a) relevance to CVD risk assessment and improved adherence to guideline recommendations. Increased trends of Lp(a) testing have been reported in several experiences from around the world (25–29). Bhatia et al. analyzed medical records at the University of California San Diego Health and reported a > 5-fold increase in Lp(a) testing between 2010 and 2020 (28). In another study by Kelsey et al. of 11 United States health systems participating in the National Patient-Centered Clinical Research Network, Lp(a) testing increased by 60.4% over 2015–2019 [3,295–5,285 Lp(a) tests] (27).

Our population was relatively young, with a low burden of AS and PVD, but with a considerably high burden of CAD. It's well-established that patients with CAD in the Middle East feature younger age at presentation (12). Finally, the observed high prevalence of any CVD, CAD, stroke, HF, and carotid stenosis among our patients with abnormally elevated Lp(a) aligns with findings of previous studies reporting a 5-fold risk of CAD, 1.7-fold risk of carotid stenosis, 1.6-fold risk of ischemic stroke, and 1.5- to 2-fold risk of HF in individuals with the highest vs. lowest Lp(a) concentrations (1).

## Limitations

Our study had several limitations, including being a retrospective single-center study. Hence, the aim of the study was to investigate resource utilization and findings of Lp(a) testing and associated CVD prevalence; data on patients' ethnicity, risk factors, medications, and other laboratory values were not collected as part of the study protocol and patients with other causes of Lp(a) elevation were not excluded from the analysis. Additionally, follow-up data on patient outcomes, including the incidence of atherosclerotic CVD or major fetal or non-fetal adverse cardiac events, were not studied. In addition, the current findings should be interpreted with caution due to potential selection bias, which may have arisen from the physician's decision-making process regarding whom to test for Lp(a). Nevertheless, this is a large series of Lp(a) tests in the UAE and Gulf region using a standardized Lp(a) assay.

## Conclusion

Almost one in six tested patients for Lp(a) had abnormally elevated Lp(a) levels and CVD was prevalent in one-third of the patients who tested abnormal for Lp(a), with one in four patients presenting with a history of CAD. The study highlights the growing awareness of the relevance of Lp(a) for CVD risk stratification and prevention, which was translated into increased testing and a higher yield of abnormal Lp(a) over time.

## Data Availability

The raw data supporting the conclusions of this article will be made available by the authors, without undue reservation.

## References

[1] Wilson DP Jacobson TA Jones PH Koschinsky ML McNeal CJ Nordestgaard BG Use of lipoprotein (a) in clinical practice: a biomarker whose time has come. A scientific statement from the national lipid association. J Clin Lipidol. (2019) 13:374–92. 10.1016/j.jacl.2019.04.010 31147269

[2] Reyes-Soffer G Yeang C Michos ED Boatwright W Ballantyne CM. High lipoprotein (a): actionable strategies for risk assessment and mitigation. Am J Prev Cardiol. (2024) 18:100651. 10.1016/j.ajpc.2024.100651 38646021 PMC11031736

[3] Craig WY Neveux LM Palomaki GE Cleveland MM Haddow JE. Lipoprotein (a) as a risk factor for ischemic heart disease: metaanalysis of prospective studies. Clin Chem. (1998) 44:2301–6. 10.1093/clinchem/44.11.2301 9799757

[4] Danesh J Collins R Peto R. Lipoprotein (a) and coronary heart disease: meta-analysis of prospective studies. Circulation. (2000) 102:1082–5. 10.1161/01.CIR.102.10.1082 10973834

[5] Nave AH Lange KS Leonards CO Siegerink B Doehner W Landmesser U Lipoprotein (a) as a risk factor for ischemic stroke: a meta-analysis. Atherosclerosis. (2015) 242:496–503. 10.1016/j.atherosclerosis.2015.08.021 26298741

[6] Kamstrup PR Tybjærg-Hansen A Nordestgaard BG. Genetic evidence that lipoprotein (a) associates with atherosclerotic stenosis rather than venous thrombosis. Arterioscler Thromb Vasc Biol. (2012) 32:1732–41. 10.1161/ATVBAHA.112.248765 22516069

[7] Kamstrup PR Nordestgaard BG. Elevated lipoprotein (a) levels, LPA risk genotypes, and increased risk of heart failure in the general population. JACC Heart Fail. (2016) 4:78–87. 10.1016/j.jchf.2015.08.006 26656145

[8] Langsted A Kamstrup PR Nordestgaard BG. High lipoprotein (a) and high risk of mortality. Eur Heart J. (2019) 40:2760–70. 10.1093/eurheartj/ehy902 30608559

[9] Langsted A Nordestgaard BG Kamstrup PR. Elevated lipoprotein (a) and risk of ischemic stroke. J Am Coll Cardiol. (2019) 74:54–66. 10.1016/j.jacc.2019.03.524 31272552

[10] Halperin JL Levine GN Al-Khatib SM Birtcher KK Bozkurt B Brindis RG Further evolution of the ACC/AHA clinical practice guideline recommendation classification system: a report of the American College of Cardiology/American Heart Association Task Force on clinical practice guidelines. Circulation. (2016) 133:1426–8. 10.1161/CIR.0000000000000312 26399660

[11] Malekpour M-R Abbasi-Kangevari M Ghamari S-H Khanali J Heidari-Foroozan M Moghaddam SS The burden of metabolic risk factors in north Africa and the Middle East, 1990–2019: findings from the global burden of disease study. EClinicalMedicine. (2023) 60:102022. 10.1016/j.eclinm.2023.102022 37287869 PMC10242634

[12] Manla Y Almahmeed W. The pandemic of coronary heart disease in the Middle East and north Africa: what clinicians need to know. Curr Atheroscler Rep. (2023) 25:543–57. 10.1007/s11883-023-01126-x 37615785 PMC10471667

[13] Bader F Manla Y Ghalib H AlMatrooshi N Khaliel F Skouri H. Advanced heart failure therapies in the eastern Mediterranean region: current status, challenges, and future directions. Curr Probl Cardiol. (2024) 49(7):102564. 10.1016/j.cpcardiol.2024.102564 38599561

[14] Alhuneafat L Al Ta’ani O Jabri A Tarawneh T ElHamdan A Naser A Cardiovascular disease burden in the Middle East and North Africa region. Curr Probl Cardiol. (2023) 49(3):102341. 10.1016/j.cpcardiol.2023.102341 38103814

[15] Mansouri A Khosravi A Mehrabani-Zeinabad K Kopec JA Adawi KII Lui M Trends in the burden and determinants of hypertensive heart disease in the eastern Mediterranean region, 1990–2019: an analysis of the global burden of disease study 2019. EClinicalMedicine. (2023) 60:102034. 10.1016/j.eclinm.2023.102034 37396799 PMC10314131

[16] Sadeghi M Jamalian M Mehrabani-Zeinabad K Turk-Adawi K Kopec J AlMahmeed W The burden of ischemic heart disease and the epidemiologic transition in the eastern Mediterranean region: 1990–2019. PLoS One. (2023) 18:e0290286. 10.1371/journal.pone.0290286 37669274 PMC10479892

[17] Mensah GA Fuster V Murray CJL Roth GA, Collaborators GB of CD and R. Global burden of cardiovascular diseases and risks, 1990–2022. J Am Coll Cardiol. (2023) 82:2350–473. 10.1016/j.jacc.2023.11.007 38092509 PMC7615984

[18] Paré G Çaku A McQueen M Anand SS Enas E Clarke R Lipoprotein (a) levels and the risk of myocardial infarction among 7 ethnic groups. Circulation. (2019) 139:1472–82. 10.1161/CIRCULATIONAHA.118.034311 30667276

[19] Kronenberg F Mora S Stroes ESG Ference BA Arsenault BJ Berglund L Lipoprotein (a) in atherosclerotic cardiovascular disease and aortic stenosis: a European atherosclerosis society consensus statement. Eur Heart J. (2022) 43:3925–46. 10.1093/eurheartj/ehac361 36036785 PMC9639807

[20] Koschinsky ML Bajaj A Boffa MB Dixon DL Ferdinand KC Gidding SS A focused update to the 2019 NLA scientific statement on use of lipoprotein (a) in clinical practice. J Clin Lipidol. (2024) 18(3):e308–e319. 10.1016/j.jacl.2024.03.001 38565461

[21] Grundy SM Stone NJ Bailey AL Beam C Birtcher KK Blumenthal RS 2018 AHA/ACC/AACVPR/AAPA/ABC/ACPM/ADA/AGS/APhA/ASPC/NLA/PCNA guideline on the management of blood cholesterol: a report of the American College of Cardiology/American Heart Association Task Force on clinical practice guidelines. Circulation. (2019) 139:e1082–143. 10.1161/CIR.0000000000001172 30586774 PMC7403606

[22] Mach F Baigent C Catapano AL Koskinas KC Casula M Badimon L 2019 ESC/EAS guidelines for the management of dyslipidaemias: lipid modification to reduce cardiovascular risk: the task force for the management of dyslipidaemias of the European Society of Cardiology (ESC) and European atherosclerosis society (EAS). Eur Heart J. (2020) 41:111–88. 10.1093/eurheartj/ehz455 31504418

[23] Pearson GJ Thanassoulis G Anderson TJ Barry AR Couture P Dayan N 2021 Canadian cardiovascular society guidelines for the management of dyslipidemia for the prevention of cardiovascular disease in adults. Can J Cardiol. (2021) 37:1129–50. 10.1016/j.cjca.2021.03.016 33781847

[24] Handelsman Y Jellinger PS Guerin CK Bloomgarden ZT Brinton EA Budoff MJ Consensus statement by the American association of clinical endocrinologists and American College of endocrinology on the management of dyslipidemia and prevention of cardiovascular disease algorithm–2020 executive summary. Endocr Pract. (2020) 26:1196–224. 10.4158/CS-2020-0490 33471721

[25] Stürzebecher PE Schorr JJ Klebs SHG Laufs U. Trends and consequences of lipoprotein (a) testing: cross-sectional and longitudinal health insurance claims database analyses. Atherosclerosis. (2023) 367:24–33. 10.1016/j.atherosclerosis.2023.01.014 36764050

[26] Bhatia HS Hurst S Desai P Zhu W Yeang C. Lipoprotein (a) testing trends in a large academic health system in the United States. J Am Heart Assoc. (2023) 12:e031255. 10.1161/JAHA.123.031255 37702041 PMC10547299

[27] Kelsey MD Mulder H Chiswell K Lampron ZM Nilles E Kulinski JP Contemporary patterns of lipoprotein (a) testing and associated clinical care and outcomes. Am J Prev Cardiol. (2023) 14:100478. 10.1016/j.ajpc.2023.100478 37025553 PMC10070377

[28] Bhatia HS Ma GS Taleb A Wilkinson M Kahn AM Cotter B Trends in testing and prevalence of elevated lp (a) among patients with aortic valve stenosis. Atherosclerosis. (2022) 349:144–50. 10.1016/j.atherosclerosis.2022.01.022 35144769 PMC9674369

[29] Zafrir B Aker A Saliba W. Lipoprotein (a) testing in clinical practice: real-life data from a large healthcare provider. Eur J Prev Cardiol. (2022) 29:e331–3. 10.1093/eurjpc/zwac124 35707956

